# A Method for High-Frequency Mechanical Scanning Ultrasonic Flow Imaging with Motion Compensation

**DOI:** 10.3390/diagnostics13081467

**Published:** 2023-04-18

**Authors:** Jiaming Heng, Chenxi Li, Tianxiang Chu, Yiwen Xu, Xiaohua Jian

**Affiliations:** 1 School of Biomedical Engineering (Suzhou), University of Science and Technology of China, Suzhou 215000, China; 2 Suzhou Institute of Biomedical Engineering and Technology, Chinese Academy of Sciences, Suzhou 215163, China; 3 School of Energy and Power Engineering, Nanjing University of Science and Technology, Nanjing 210094, China

**Keywords:** color flow imaging, high-frequency ultrasound, mechanical scanning, Doppler

## Abstract

Mechanical scanning with a single transducer is an alternative method for high-frequency ultrasound imaging, which is simple in structure, convenient to implement, and low in cost. However, traditional mechanical scanning ultrasonic imaging introduces additional Doppler shift due to the movement of the transducer, which brings a challenge for blood velocity measurement. An improved mechanical scanning system for high-frequency ultrasonic color Doppler flow imaging is developed in this paper. The mechanical scanning system has a scanning stroke range of 15 mm, a maximum scanning speed of 168 mm/s, and an imaging depth of 20 mm. Since the mechanical scanning of the system is not in uniform motion, motion compensation was applied to achieve high-precision imaging both in B-mode and Doppler mode. The experiment results show that the system imaging resolution can reach about 140 μm in B-mode imaging, the relative velocity error is less than 5% in color Doppler flow imaging at different flow rates, and the CNR of power Doppler flow imaging of this system is greater than 15 dB. The proposed mechanical scanning imaging system can achieve high-resolution structure imaging and color flow imaging, which can provide more diagnostic information for the practical diagnosis and broaden the application range of mechanical scanning ultrasound imaging.

## 1. Introduction

High-frequency ultrasound imaging has been increasingly gaining development in recent years due to its high-resolution advantages, which can provide more detail and information of the diseased tissues/organs to improve the accuracy of clinical diagnosis and treatment [1,2]. For example, intravascular ultrasound imaging can clearly differentiate plaques and the stenosis of carotid arteries, providing a powerful basis for pre- and post-stent placement evaluation [3]. Endoscopic ultrasound imaging, including digestive endoscopic ultrasound and bronchoscopy ultrasound, can effectively identify the size and infiltration degree of tumors, providing a basis for tumor classification [4]. In addition, ophthalmic ultrasound imaging, skin ultrasound imaging, and high-frequency ultrasound imaging for small animals have been increasingly used in clinical and scientific research, providing a strong method for the fine diagnosis of related diseases and theoretical research [5]. However, high-frequency ultrasound imaging has high performance requirements for the system, requiring higher sampling rates, faster processing speeds, and high-performance ultrasound probes. Especially for traditional multi-channel high-frequency ultrasound imaging systems, 128–256 working channels are usually required, but at present, the performances of front-end multi-channel transmission stimulation chips, multi-channel high-frequency sampling chips, etc. are extremely limited. In addition, the production of multi-element high-frequency ultrasound probes is difficult, complex, and the cost is high. These have greatly limited the popularization and application of high-frequency ultrasound imaging.

The mechanical scanning high-frequency ultrasound imaging system is a feasible high-frequency ultrasound imaging method [6]. It usually uses a mechanical scanning structure to drive a small high-frequency ultrasound transducer to reciprocate and achieve real-time imaging of the target. Since only one ultrasound transducer element and one working channel are required, the mechanical scanning high-frequency ultrasound imaging system is simple in structure, easy to manufacture, and has been widely used in recent years. For example, mechanical intravascular ultrasound imaging is achieved by utilizing a miniature high-frequency transducer at the tip of a catheter that rotates 360 degrees [7]. Ophthalmic ultrasound imaging using mechanical scanning is achieved by reciprocally fan-scanning the transducer inside the probe to achieve high-resolution imaging of the eyeball [8]. There is also a method of keeping the transducer stationary and driving the reflecting mirror to achieve high-frequency scanning imaging of the target [9].

In addition, Doppler ultrasonography can display blood flow information inside tissues in real-time, which is an important diagnostic tool in the diagnosis and treatment of various diseases [10,11]. For example, carotid artery ultrasound Doppler can be used to diagnose the narrowing of blood vessels and plaque in the neck, and stroke risk [12]. Transcranial Doppler can obtain the hemodynamic parameters of the cerebral basilar artery to reflect the functional state of the cerebral vascular [13]. At present, ultrasound Doppler can be mainly divided into spectral Doppler and 2D Doppler imaging. Typical representatives of 2D Doppler imaging are color Doppler and power Doppler. Color Doppler overlays the velocity information of the blood corpuscle over a B-Mode image, which is useful to interrogate organs for the presence or absence of blood flow. Meanwhile, power Doppler overlays the energy of blood Doppler signal instead of frequency onto a B-mode image, which can effectively increase the sensitivity for detecting blood flow in low flow states. Therefore, both Color Doppler and power Doppler modes of the system are proposed in our study. However, the current research of high-frequency ultrasonic Doppler imaging technology is mainly based on multi-channel ultrasound imaging systems and liner probes [14,15], while the current mechanical scanning high-frequency ultrasound imaging is mainly focused on high-resolution B-mode imaging and there is a lack of research on ultrasound blood flow imaging [16]. This is mainly due to the higher requirements of the transmission frequency and processing speed of the mechanical scanning imaging system for ultrasound Doppler imaging. In addition, due to the relative motion between the mechanical scanning motion and the tested blood flow, the mechanical scanning imaging itself will bring corresponding errors to the measurement results. Although some high-frequency mechanical scanning ultrasound systems were used to measure blood flow parameters, only the blood flow velocity at a specified location is calculated [17,18]. In addition, although existing single-channel high-frequency ultrasound systems can also use a displacement platform or piezoelectric motors for point-by-point scanning for blood flow imaging [19], the imaging is very slow and their mechanical scanning system or probe are bulky and unsuitable for clinical applications. Therefore, we designed and developed a high-performance mechanical scanning imaging system, which meets the requirements of ultrasound Doppler imaging in real-time and with a suitable size, takes into account the non-uniform motion errors in the mechanical scanning process, and performs corresponding motion compensation. The accuracy of the relevant theoretical analysis and imaging system was verified using a blood flow phantom; finally, high-accuracy ultrasound blood flow imaging using power Doppler and color Doppler was achieved.

## 2. Materials and Methods

### 2.1. System

Our high-frequency mechanical scanning ultrasound imaging system is mainly composed of three parts: mechanical scanning structure, high-frequency ultrasound probe, and imaging system.

#### 2.1.1. Mechanical Scanning Module

**Figure 1:**
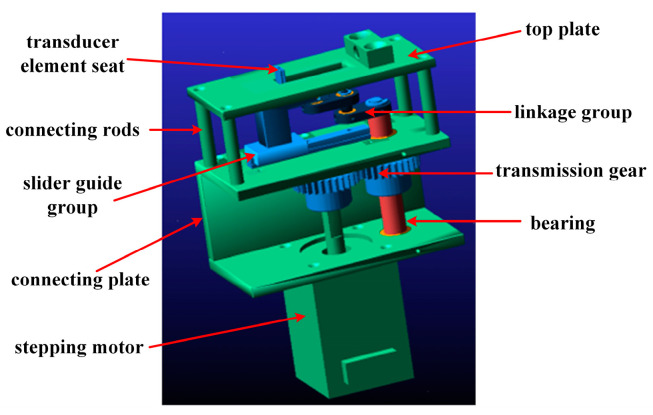
The schematic of the mechanical scanning module.

A mechanical scanning structure is mainly implemented to achieve linear reciprocating movement of the micro high-frequency transducer to produce imaging that can reach the imaging effect of traditional ultrasound linear array/phased array transducers. The mechanical scanning module of this system converts rotary motion into linear motion through a connecting rod mechanism. The connecting rod mechanism is composed of several components with a definite relative motion connected by low pairs, including a rotary pair and moving pair. Due to the low pairs being surface-contacting and wear-resistant, and the contact surfaces of the rotary pair and moving pair being cylindrical and planar, they are easy to manufacture with a high degree of accuracy. The specific structure of the mechanical scanning system is shown in Figure 1. Its main components include a stepping motor (SUMTOR 20HS2806A4), transmission gear of the same level, transmission shaft, bearing, sleeve, cotter, slider guide group (THK RSR3M), crank, connecting rods, spring, support seat, transducer element seat, motor fixing plate, connecting plate, connecting columns, slide rail fixing plate, and a top plate. The mechanical scanning system has a scanning stroke range of 15 mm and a maximum scanning speed of 168 mm/s. Compared with the traditional gear and rack mechanism, the stepping motor does not need to switch between forward and reverse in our solution and can rotate continuously to achieve a continuous linear reciprocating motion of the transducer, which helps to improve the frame rate of the system imaging.

However, it must also be noted that the disadvantage of this mechanical structure is that the crank connecting rod mechanism has a rapid return characteristic and its motion state is nonlinear, which affects the accuracy of ultrasound imaging. It is also the reason for the need for motion compensation.

#### 2.1.2. High-Frequency Transducer

**Figure 2:**
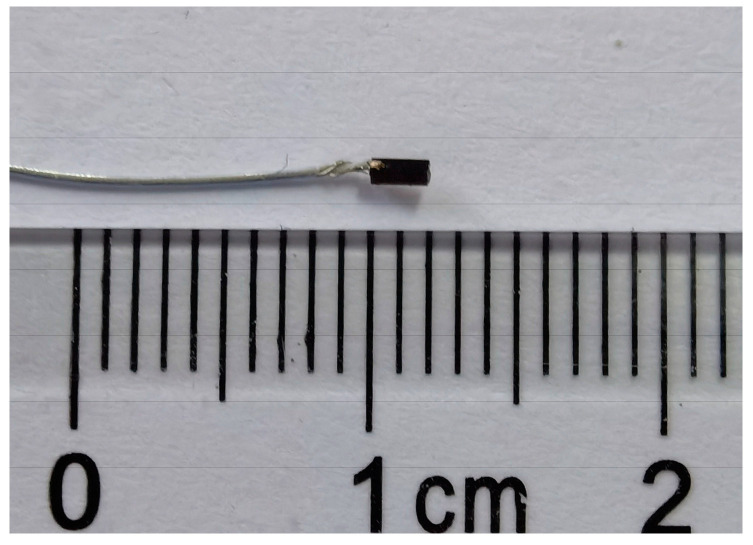
The fabricated micro high-frequency transducer.

A small high-frequency transducer was designed and fabricated for the mechanical scanning imaging system, as shown in Figure 2. Considering the mounting fixation and imaging performance, the transducer size was designed to be 2.0 × 0.7 mm with a center frequency of 12 MHz. This operating frequency ensures that the transducer has both high imaging resolution (~150 μm) and deep imaging depth (~20 mm) to enable the detection of deeper blood vessels under the skin. The transducer consists of a 130 μm PZT piezoelectric layer, a 0.9 mm silver epoxy backing layer, and a thin matching layer. The detailed fabrication process can be found in our previous publications [20,21]. The transducer was connected to the system using a 42 AWG coaxial cable.

**Figure 3:**
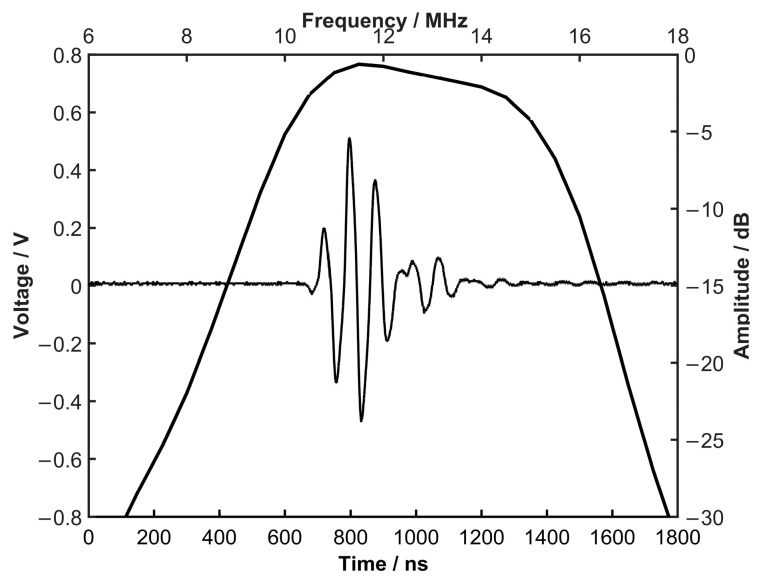
The measured echo and frequency wave of the fabricated transducer.

The pulse echo of the fabricated high-frequency transducer was measured with a DPR500 pulser–receiver (JSR Ultrasonics, Pittsford, NY, USA). A flat Ackley plastic plate placed 5 mm away from the transducer was used as the reflective target. In Figure 3, the black line is the detected pulse–echo waveform, and the blue line is the calculated frequency spectrum by performing a Fourier transform on the echo data. According to the results, the fabricated transducer has a center frequency of 12.6 MHz, a bandwidth @−6 dB of 48.2%, and a peak-to-peak value of 984 mV, meeting the requirements of high-frequency mechanical scanning ultrasound imaging.

#### 2.1.3. Mechanical Scanning Imaging System

**Figure 4:**
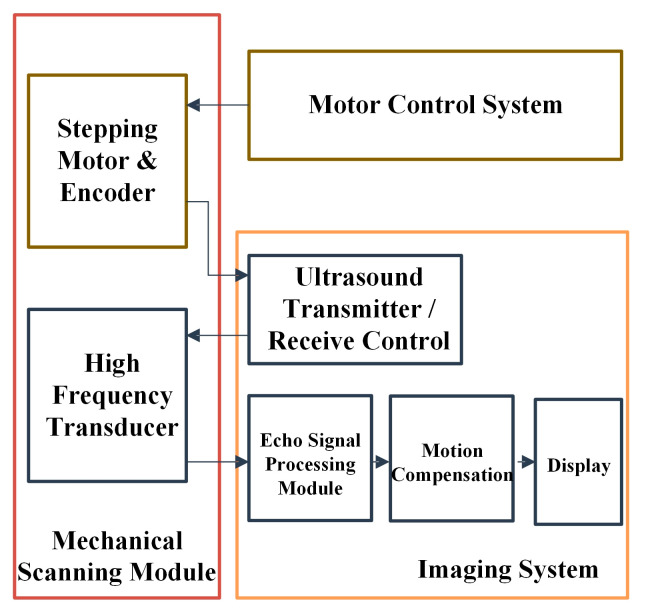
The schematic diagram of the mechanical scanning ultrasound imaging system.

The schematic of the mechanical scanning imaging system is shown in Figure 4. The entire experimental system consists of a mechanical scanning module, a motor control module, and an imaging platform. The core mechanical scanning module contains the abovementioned mechanical scanning part, a high-frequency transducer, a drive motor, and an encoder. The overall dimensions of the mechanical module are 5 cm × 2.2 cm × 12 cm, which makes it possible to later package it into a handheld probe. The basic principle of the system is to use the high-precision mechanical scanning module to drive the single-element high-frequency transducer for real-time, high-resolution B-mode and color Doppler flow imaging. The echo signals received by the transducer were post-processed by a high-frequency Vantage system (Verasonics, Redmond, WA, USA).

**Figure 5:**
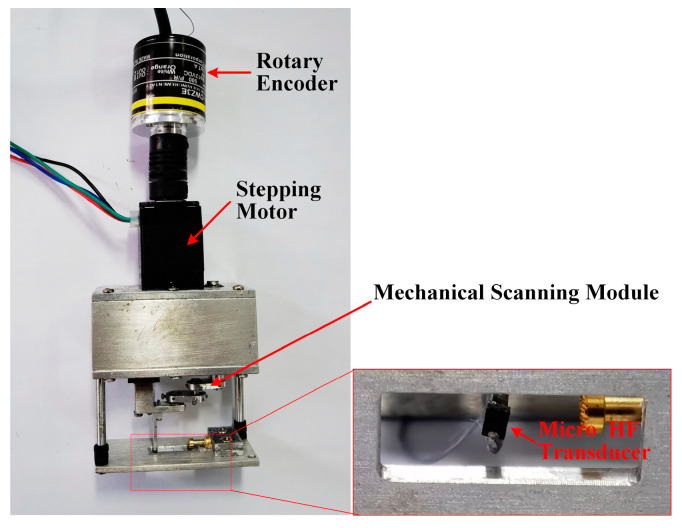
The fabricated mechanical scanning system.

A 500 P/R encoder (20HS2806A4, Omron) was used to record the rotation position of the motor in real time to obtain the precise positioning of the transducer. Because the stepping motor rotates 1 turn and the transducer is scanned reciprocating once, the mechanical scanning positioning accuracy of the system can reach 60 μm. The fabricated mechanical scanning imaging system is shown in Figure 5. In the experiment, both the imaging target (tungsten wire phantom or blood flow phantom) and the front end of the mechanical scanning module (mainly the transducer part) were immersed in a sink to test the relevant imaging performance. Regarding the operating temperature of the system, because its application goal is for clinical diagnosis, the experiments were carried out at room temperature. Tested by sink heating below 40 degrees Celsius, since the mechanical scanning module and transducer have good temperature resistance, the performance of the system was very stable.

### 2.2. Method

#### 2.2.1. Ultrasound Doppler Flow Imaging Method

**Figure 6:**
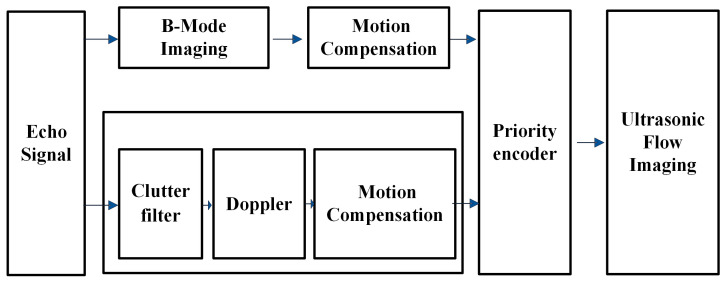
The processing flowchart of ultrasonic flow imaging.

The processing flow of the ultrasonic imaging system is shown in Figure 6. After the ultrasonic echo signals were filtered by clutter filtering, the pulsed-wave Doppler blood flow signals were extracted; finally, the blood flow images were overlaid on the B-mode images by color coding to form the ultrasonic blood flow imaging. Both the B-mode images and the blood flow imaging images were motion compensated to achieve correct imaging, and the specific analysis can be seen in the motion compensation chapter. Due to the fact that the blood flow information received by the system contains not only blood flow signals but also clutter signals (mainly generated by the motion of the vessel wall and its surrounding tissues) and system noise signals, in order to obtain a high signal-to-noise ratio of the blood flow signals, clutter filtering of the echo signals must be performed first. Considering the filtering effect and measurement speed, the regression polynomial based on the least squares method was used for filtering the main component of the blood flow signal in the area of blood flow, and the clutter filter based on the Hankel singular value decomposition was used for filtering the clutter in the non-bloodstream areas [22,23]. After filtering, the echo signals were used to extract the blood flow parameters such as signal magnitude, velocity, and direction of the blood flow by the pulsed-wave Doppler principle and the autocorrelation algorithm [24]. Combined with the relative scanning speed of the transducer, the blood flow information was motion compensated accordingly. Finally, the ultrasonic blood flow imaging was completed by a priority encoder and combined with the B-mode images.

The system uses two blood flow imaging algorithms, power Doppler and color flow Doppler. Among them, power Doppler imaging is used to image the extracted blood flow power information, which can provide higher blood flow imaging sensitivity and is suitable for detecting vessels in deeper and internal organs. Color flow Doppler imaging is a blood flow imaging of the extracted blood flow velocity information, which can quantify the speed, direction, and other information of blood flow, and provide a basis for accurate diagnosis and treatment.

#### 2.2.2. Mechanical Scanning Motion Compensation

**Figure 7:**
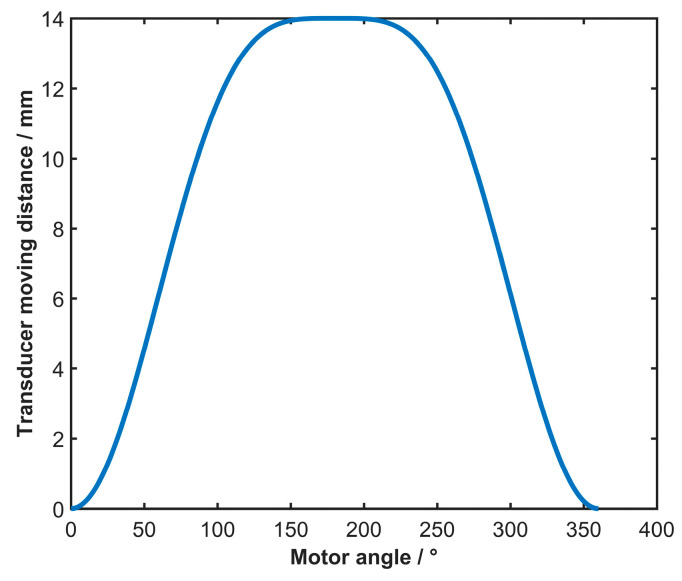
The movement trace of the mechanical scanning.

Since the mechanical scanning of the system is not in uniform motion, the displacement distance at each moment is different when the stepping motor rotates in one round. According to the transmission structure, the relationship between the horizontal movement distance of the transducer and the rotation angle of the motor (obtained by the encoder) was calculated as shown in Figure 7. The motion trajectory as a whole presents a sinusoidal change, and its motion in the middle region is approximately linear, but at its top it has a significant hysteresis in the range of motor angles from 152° to 208°, which is mainly caused by the mechanical scanning method of the connecting rod drive. This nonlinear motion will result in errors in imaging target distortion and blood flow velocity. Therefore, motion compensation for its high-precision imaging is essential.

**Figure 8:**
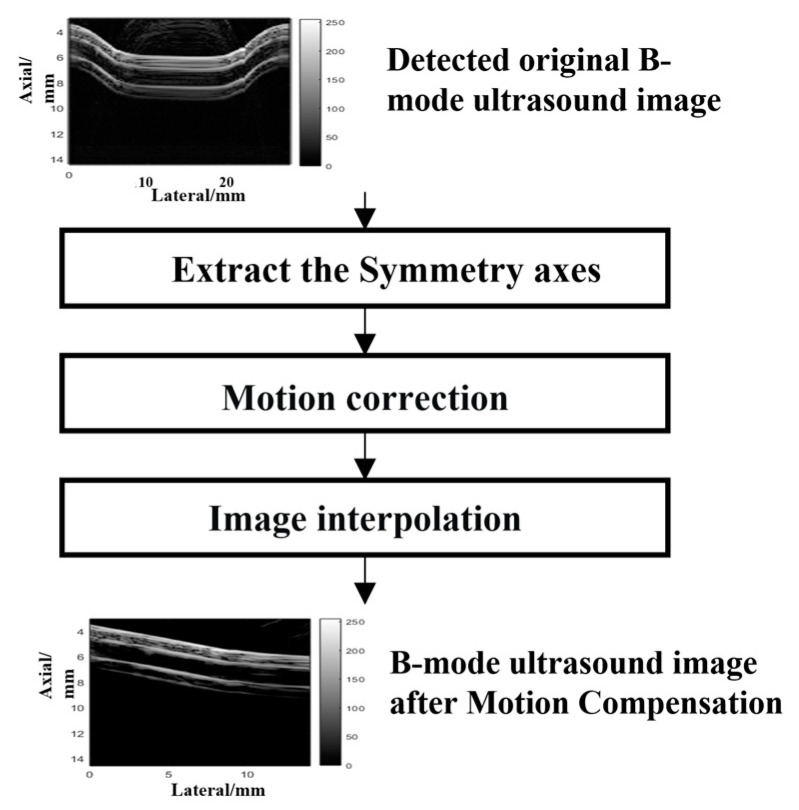
Schematic diagram of the process of motion compensation.

The process of motion compensation is shown in Figure 8. Before the motion compensation, the starting point of the scanning motion and the position of the maximum displacement point were first found through similarity calculation. The image was then motion-corrected by a motion relationship curve, as shown in Figure 7. Considering the difference in spacing between the motion-corrected data, the corrected data were interpolated three times by Hermite interpolation to achieve the correct image.

Specifically, the motion compensation mainly consists of two parts: The first part is in B-mode imaging—according to the motion trajectory, the spatial position of the obtained echo raw data is adjusted to ensure that the target does not present obvious image distortion. The other part is that when Doppler calculates, the real-time motion speed of the transducer is not processed at a uniform speed but the obtained blood flow velocity is corrected at different positions according to the results of Figure 7.

### 2.3. Phantom

#### 2.3.1. Wire Phantom

**Figure 9:**
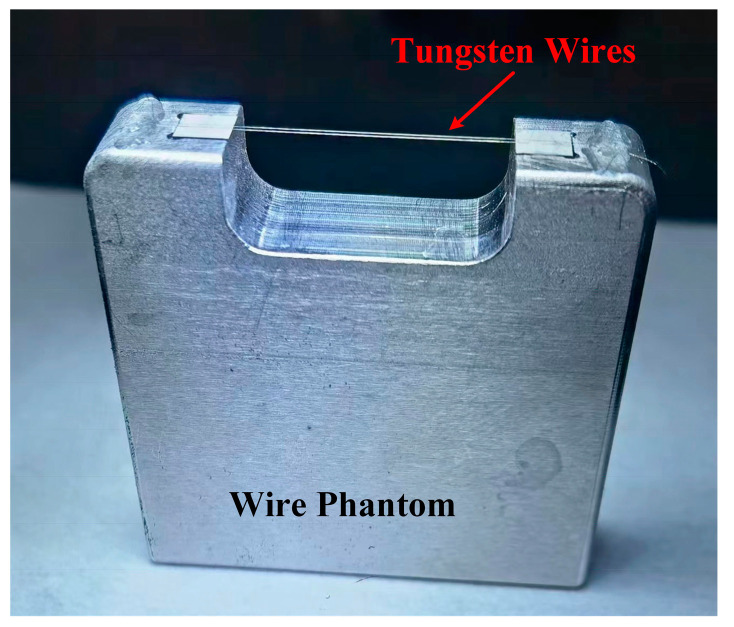
The tungsten wire phantom.

Wire phantoms were primarily used to test the B-mode imaging resolution of the developed system. The wire target mimicry consisted of two tungsten wires with a diameter of 10 μm and a spacing of 500 μm, as shown in Figure 9. The two tungsten wires were bonded to a 4 cm long stainless steel base for easy movement and use. When testing, the wire phantom was placed 8–10 mm away from the transducer to simulate superficial imaging.

#### 2.3.2. Flow Phantom

**Figure 10:**
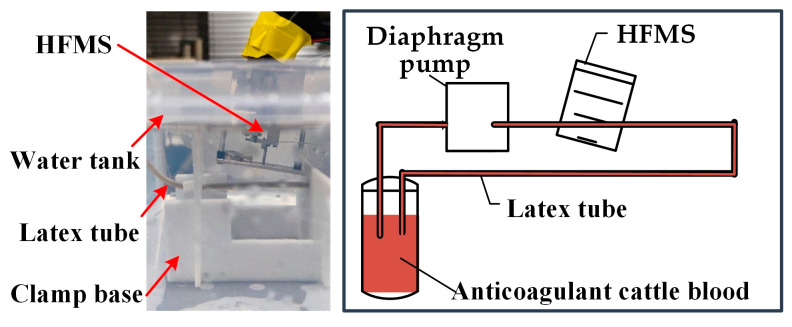
The flow phantom photos and schematic diagram.

The flow phantom is mainly used to test the blood flow imaging performance of development systems. The hemodynamic parameters of the radial artery near the radius were referred to in the flow phantom experiment [25]: blood vessel diameter 2.07 ± 0.32 mm, wall thickness 0.39 ± 0.08 mm, systolic peak flow rate 51 ± 15 cm/s, and blood vessel depth less than 10 mm. Figure 10 shows the built flow phantom and test device. Among them, anticoagulant cattle blood was selected as imitation blood, and a latex tube with a diameter of 2 mm and a wall thickness of 0.5 mm was used as a blood vessel imitation. The latex tube was held in place by a custom 3D-printed scaffold, and the angle of detection was adjusted by controlling the scaffold and system probe. According to the detection needs, the angle between the blood vessel imitation and the system HFMS was set as 10° and the distance was about 5 mm.

To simulate the blood flow movement at different flow rates of 20~70 cm/s, three different diaphragm pumps were used, namely, the diaphragm pump with adjustable flow rate KPV04 and the diaphragm pumps KLC2-A and EDVP083 with non-adjustable flow rates. The method of measuring the blood flow rate in phantom is as follows: first, 10 mL of bovine blood was measured by a pipette; the measured weight was 10.40 g and the density of bovine blood was calculated as 1.04 g/cm^3^. Then, the weight of blood pumped by the diaphragm pump was measured for half a minute; from this weight, the flow of liquid from the diaphragm pump per minute could be calculated. Finally, the average flow rate of blood flow *V* within the latex tube with an inner diameter of 2 mm was calculated as shown in the following equation:

(1) $$v=\frac{m}{60\pi r^{2}\rho }$$

where *m* is the mass of bovine blood, ρ is the density of bovine blood, and *r* is the inner diameter of the mimetic blood vessel.

Furthermore, according to the flow phantom parameters and pulse-wave Doppler flow parameter formula, the pulse repetition frequency of the system at different flow rates was determined. The parameters of the blood flow phantom experiment setup at different flow rates are shown in Table 1.

## 3. Results

### 3.1. Wire Phantom Imaging

**Figure 11:**
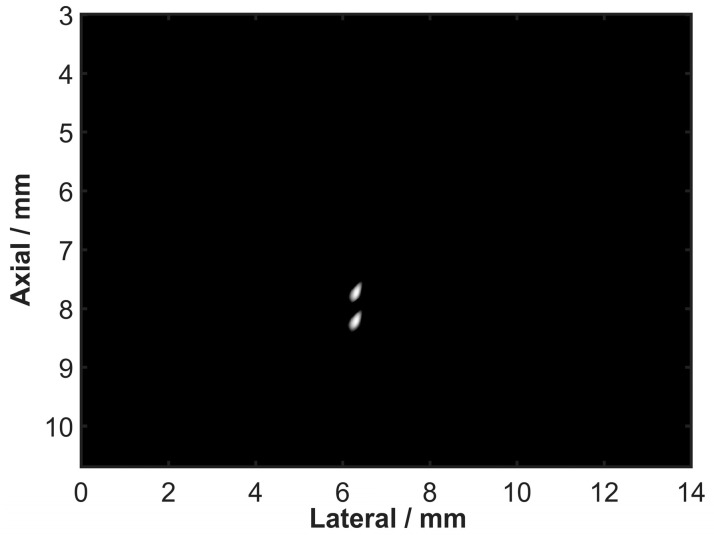
The detected wire phantom image.

Firstly, the imaging resolution of the mechanical scanning ultrasound imaging system was tested by wire phantom experiment. The detected wire phantom imaging results are shown in Figure 11, where the two wires are clearly visible.

**Figure 12:**
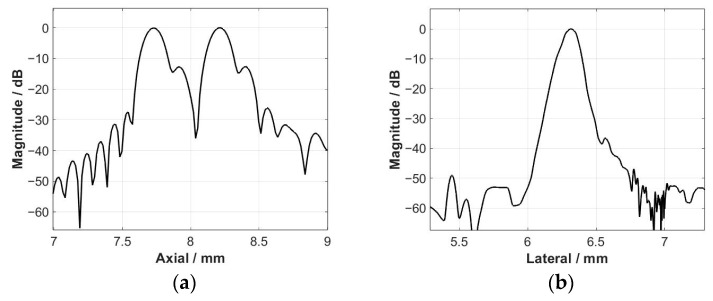
(**a**) The captured axial beam profiles and (**b**) lateral beam profiles.

The full width at half maximum (FWHM) of the envelope signals of the wire was taken as a characterization parameter for system lateral and axial imaging resolution, as shown in Figure 12 below. According to evaluation of the line spread function based on Rayleigh Criterion [26,27], the lateral and axial resolutions of the system are 140 μm and 168 μm, respectively.

### 3.2. Flow Phantom Imaging

**Figure 13:**
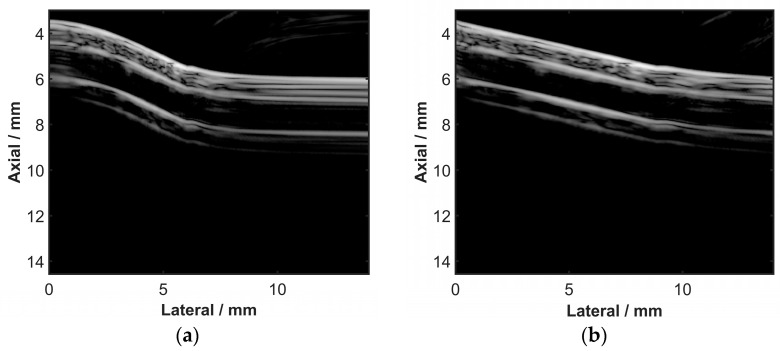
The detected B mode images of the flow phantom: (**a**) the original image without motion compensation and (**b**) the compensated image.

First, the system was used to perform B-mode imaging of the vessel phantom, as shown in Figure 13. It can be seen that the non-motion-compensated image (Figure 13a) is significantly distorted in the regions at both ends of the image compared with the motion-compensated image (Figure 13b). As expected, imaging with motion compensation can effectively improve the imaging accuracy of the target structure and the accuracy of subsequent measurements.

**Figure 14:**
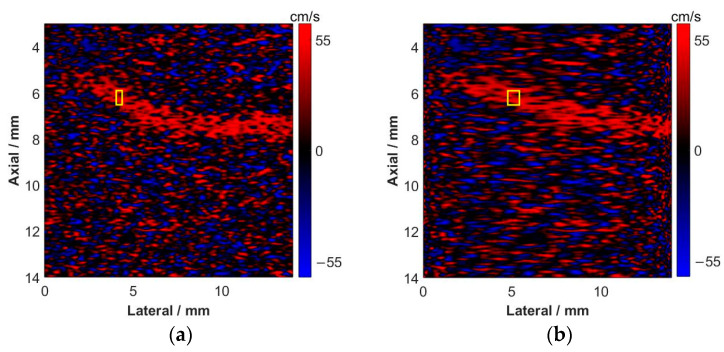
Flow image information calculated with color flow Doppler: (**a**) original image without motion compensation and (**b**) the compensated image. The yellow rectangular area was used to measure the average blood velocity.

**Figure 15:**
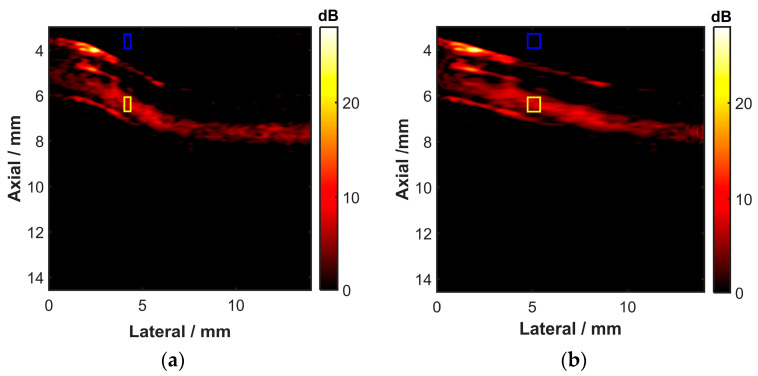
Flow image information calculated with power Doppler: (**a**) original image without motion compensation and (**b**) the compensated image. The blood flow area (yellow rectangular area) and the clutter area (blue rectangular area) were used to calculate the CNR.

The flow imaging of the phantom was then performed using the system. First, the color flow Doppler imaging method was applied to obtain the blood flow velocity information of the phantom, as shown in Figure 14. According to the results, the average blood velocity measured before motion compensation was 42.2 cm/s. Since the actual blood flow of the phantom is 44 cm/s, the measurement error is about 4.12%. After motion compensation, the average blood velocity was 42.2 cm/s and the error was reduced to 4.04%. At the same time, in the color hemography, the blood flow direction shown is consistent with the actual flow direction. It should be noted that on the color flow Doppler figure, in addition to the area inside the blood vessels, a lot of noise is also visible in other background areas. This is mainly because the high-frequency clutter signal in the background area is retained after the algorithm is filtered but these noise assignments are small, as shown in their power spectrum (Figure 15), and can be filtered out by the power information.

Next, the power information of the mimetic blood flow was calculated, as shown in Figure 15. In the power flow image, it can be clearly seen that the energy information of blood flow is concentrated in the blood vessels. Contrast-to-Noise Ratio (CNR) was introduced to evaluate the images [28]. The CNR of the images before and after motion compensation was calculated to be 16.33 dB and 16.37 dB, respectively. It can be seen that motion compensation has limited improvements to CNR, but its position information has been further corrected by motion compensation.

**Figure 16:**
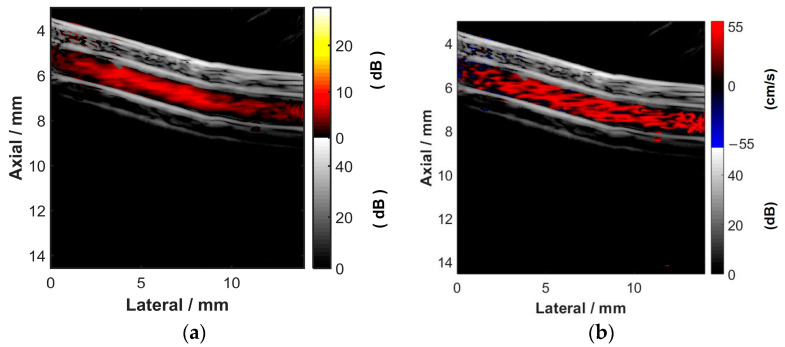
The fused color flow imaging results of the blood flow phantom with B-mode image of (**a**) power Doppler image and (**b**) color flow Doppler image.

Finally, the motion-compensated power Doppler and color flow Doppler image were fused with the above B-mode image, and the final color blood flow image is shown in Figure 16. The images clearly show the vascular phantom structure, and the blood flow information inside, which will be helpful for diagnosis.

**Figure 17:**
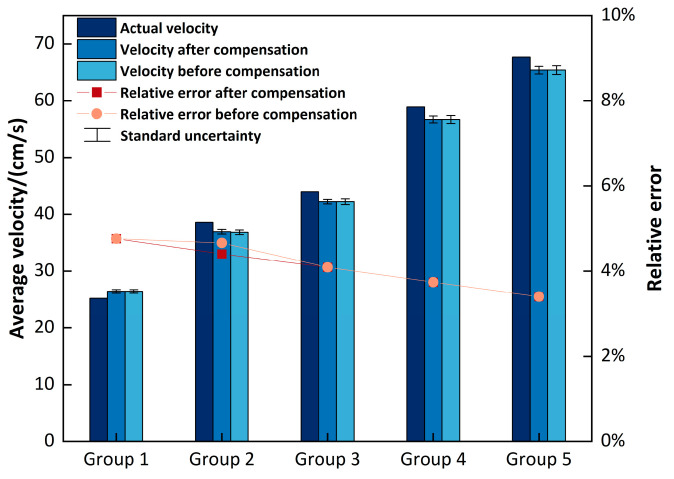
Statistical analysis of blood flow average velocity, relative error, and standard uncertainty results detected by the system at different flow rates.

The tests were repeated five times. The average flow rate and relative error results obtained by the system at different flow rates are shown in Figure 17. According to the results, after the motion compensation, the error of the system to detect the average velocity of blood flow is less than 5%. There will be a slight improvement after motion compensation compared to before motion compensation; this is because the speed measurement we chose is in the middle region of the scanned image, where the speed of the mechanical scan is linear (as shown in Figure 7) and the error is not large. However, if selected in the left and right end areas of the image, not only is the image distortion large but the motion compensation correction is also more necessary. In addition, the absolute error of the system to detect the blood flow rate is about 2 cm/s; so, the larger the flow rate, the smaller the relative error. Further, the uncertainty of the velocity measurement results was evaluated using the standard deviation formula [29,30]:

(2) $$u(x_{k})=\sqrt{\frac{\sum_{k=1}^{n}{\left(x_{k}-\bar{x}\right)}^{2}}{n-1}}$$

where $u(x_{k})$ is the standard uncertainty of measurement, $x_{k}$ is the *k* th velocity measurement result, *k* = 1, 2…, *n*, and $\bar{x}$ is the average velocity of flow rate. According to the calculated results shown in Figure 17, the standard uncertainty of the velocity is less than 1 cm/s and the deviation is less than 1% after motion compensation; so, the stability of the system measuring blood flow is good. The specific results are detailed in Table 2.

**Figure 18:**
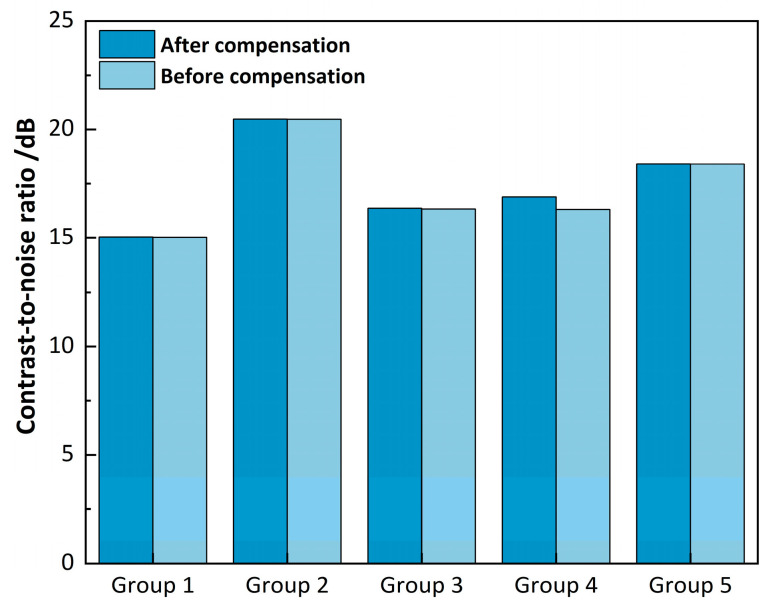
Statistical results of blood flow average CNR detected by the system at different flow rates.

In addition, the CNR results obtained by the system at different blood flow rates were statistically analyzed, as shown in Figure 18. It can be seen that through multiple groups of experiments, the CNR of power Doppler flow imaging based on mechanical scanning is greater than 15 dB, which can effectively extract the signal distribution of the blood flow region. At the same time, it can also be found that motion compensation has less influence on CNR and it is almost the same at different speeds.

Finally, the relevant data are summarized in Table 2.

## 4. Discussion

In summary, a mechanical scanning high-frequency ultrasound imaging system was developed and, based on this system, high-frequency ultrasound flow imaging was realized by Doppler flow imaging technology and motion compensation. In the wire target imaging experiment, the B-model ultrasound image can accurately display the position of the target wire; its lateral/axial imaging resolution reaches 140 μm and 160 μm, respectively; and the position accuracy error is 1.44%. The imaging resolution of the system can be expected to further improve if higher-frequency transducers and high-performance imaging systems are used. In the future, according to the needs of different application scenarios and resolutions, the high-frequency mechanical scanning imaging system can replace different probes, and this replacement is very convenient. In addition, according to the results of flow phantom experiments, the system can display the front and back walls of the blood vessels, and can realize power Doppler flow imaging and color blood flow imaging. The error of detecting the average velocity of blood flow is less than 5% at different flow rates. The CNR of power Doppler flow imaging of this system is greater than 15 dB. The results of the experiment illustrate the potential of the mechanical scanning system, which can provide multi-dimensional diagnostic information for clinical use on the basis of traditional high-frequency mechanical line scanning B-mode ultrasound imaging.

In addition, since the sound velocity of human tissues changes at different temperatures, and for human soft tissues and blood the sound velocity increases with body temperature increases [31,32], more accurate distance measurement and target imaging can be achieved by compensating and calibrating the acquired echo signal. Furthermore, the mechanical scanning probe of our system is small in size and light in structure, which can realize handheld operation, but considering that the current mechanical scanning device does not have a shell package, with the handheld it is easy to mistakenly touch the encoder and cause inaccurate measurement; so, the current experiment is still carried out by clamping to fix the probe. However, in theory, because our mechanical scanning system has a fast imaging frame rate, similar to the conventional B-ultrasound system, the slight jitter generated by the operator will not have a large impact on the final imaging result. In addition, the influence of handheld jitter can be further reduced by optimizing the scanning structure and adding fixtures.

Therefore, we believe that the developed mechanical scanning high-frequency ultrasound imaging system will further improve the application range of mechanical scanning ultrasound imaging device; is expected to play a role in vascular puncture, vascular disease diagnosis, and other fields; and has a good clinical application prospect.

## Figures and Tables

**Table 1:** Parameters of blood flow phantom experiment setup at different flow rates.

Group	Flow Rate (cm/s)	Pulse Repetition Frequency (Hz)	Experimental Diaphragm Pump Model
1	25.2	2500	KVP04-1
2	38.6	4000	EDVP08
3	44.0	3000	KLC2-A-1
4	58.9	4000	KVP04-2
5	67.7	4000	KLC2-A-2

**Table 2:** Summary of the mechanical scanning high-frequency ultrasound imaging system test results.

Group	Actual Flow Rate (cm/s)	Before Compensation					After Compensation		
		Flow Rate (cm/s)	Relative Error (%)	Standard Uncertainty (cm/s)	CNR (dB)	Flow Rate (cm/s)	Relative Error (%)	Standard Uncertainty (cm/s)	CNR (dB)
1	25.2	26.4 ± 0.3	5.03	0.3	15.02	26.4 ± 0.3	4.98	0.3	15.03
2	38.6	36.8 ± 0.4	4.75	0.4	20.46	36.9 ± 0.4	4.73	0.4	20.47
3	44.0	42.2 ± 0.5	4.12	0.5	16.33	42.2 ± 0.4	4.04	0.4	16.37
4	58.9	56.7 ± 0.7	3.81	0.7	16.31	56.7 ± 0.6	3.65	0.6	16.89
5	67.7	65.4 ± 0.8	3.38	0.8	18.41	65.5 ± 0.7	3.33	0.7	18.42

## Data Availability

The data supporting this study’s findings are available in the Database Science Data Bank(Doi: 10.57760/sciencedb.07981).
